# A Report of Three Cases With Dissociative Disorder: Some Positive Response to Methylphenidate Extended Release Used for Their Concurrent Attention Deficit Hyperactivity Disorder

**DOI:** 10.1002/npr2.70004

**Published:** 2025-02-21

**Authors:** Kenichiro Okano

**Affiliations:** ^1^ Hongounomori Clinic Bunkyo‐Ku Tokyo Japan

**Keywords:** ADHD, clinical, dissociative disorder, methylphenidate, psychopharmacology

## Abstract

Although no psychotropic medication is specifically effective for dissociative symptoms, certain abusive substances can markedly alter these symptoms. Regarding psychotropic medications, a positive response was observed in certain patients with dissociative symptoms who also have attention deficit hyperactivity disorder (ADHD), for which methylphenidate extended release (MER) was administered. A is a female in her twenties and has dissociative identity disorder (DID) and ADHD. Primary personality A1 is usually on duty but always on guard, as her other personality, A2, often attempts to disturb A1. A1's job is also hampered by careless mistakes due to her ADHD. After A was put on MER, A1 felt that A2's presence suddenly faded away while on duty and she could focus better on her job as a result. B is a middle‐aged male with DID and ADHD whose life is disturbed by the occasional appearance of a violent and disruptive personality. When MER was administered for his ADHD symptoms, it helped him stay alert, and his violent episodes decreased significantly. C is a young male university student with occasional dissociative “foggy” episodes that leave him with amnesia. He is diagnosed with depersonalization‐derealization disorder (DDD) and ADHD. After MER was administered for his ADHD symptoms, his dissociative episodes diminished markedly, and his job performance improved significantly as a result. When MER is administered for patients with dissociative conditions and comorbid ADHD, it appears to have positive effects on their dissociative symptoms, including increasing the threshold separating different personalities or diminishing depersonalization symptoms.

## Abstract 2

Three cases of dissociative identity disorder with ADHD were described. The use of Methylphenidate improved not only ADHD but also their dissociative symptoms.

## Graphical abstract

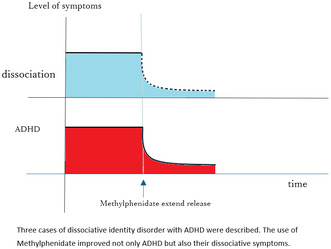

## 1 Background

Dissociative disorder is a complex, protean, and intriguing condition, the understanding and treatment of which remains controversial among clinicians. Dissociative symptoms are often unpredictable and arise suddenly, disturbing the social and daily function of individuals with this condition. However, there is a general understanding among clinicians that there is no psychotropic medication that improves dissociative symptoms [1]. Clinical observations sometimes provide a glimpse of how certain chemical agents may affect the degree and nature of dissociative conditions. Notably, some psychedelic or anesthetic agents are known to be “dissociative,” causing dissociation‐like experiences among individuals with no known dissociative disorder.

Among the many substances of abuse, alcohol appears to have a potent effect on dissociative conditions. One of my patients, a middle‐aged corporate employee with dissociative identity disorder (DID) was well adapted to his daily office work. During one of his regular clinical visits, he appeared composed as usual and reported occasional switching between different personalities. After the visit, he had a can of beer on his way home, became inebriated, and decided to return for another “small chat” with me. When I saw him again, he demonstrated rapid and uncontrollable shifts in personality. At one moment, he was in a very challenging and emotional male personality state, making some derogatory remarks, then shifted back to his usual composed main personality and apologized for the rude behavior (usually, this patient is often co‐conscious of other personalities' remarks and behaviors), only to return to his challenging and wild state. In my observations, consuming even small amounts of alcohol or benzodiazepine can significantly reduce the threshold separating different personalities in patients with DID.

In contrast, stimulant drugs appear to increase this threshold. One of my patients with DID, a female in her twenties with a history of amphetamine abuse, had occasional dissociative fugues that often disrupted her social and personal life. Her own observation was that her fugue never occurs when she is under the influence of amphetamine, and this was one of the reasons she continued to use the drug.

During my practice with dissociative patients, I became aware that certain psychotropic medications may also improve patients' dissociative conditions. A proportion of my patients with dissociative disorders also have attention deficit hyperactivity disorder (ADHD) symptoms, for which I administered methylphenidate extended release (MER, Concerta) to alleviate their ADHD symptoms. Unexpectedly, I discovered that in many of these patients, not only were their ADHD symptoms alleviated, but their dissociative symptoms also improved significantly.

Curiously, there are only a few psychiatric literatures which discuss methylphenidate or MER's effectiveness on dissociative symptoms. One case report states that a middle‐aged woman with DID and ADHD showed marked improvement in Dissociative Experiences Scale when methylphenidate was administered [2]. Two case reports discuss MER's effectiveness on depersonalization, but not on dissociative symptoms in general [3, 4].

In this report, I describe clinical examples that I encountered and discuss the potential effectiveness of MER on dissociative symptoms.

## 2 Case Presentation

A is a female in her late twenties with a diagnosis of DID. She was also diagnosed with ADHD during childhood. She has worked at a law firm for the past several years after graduating from law school. Her main personality, A1, performs her job fairly well, but because of her attentional problems, A1 often makes careless mistakes. A1's job is hampered by another personality, A2, who often attempts to take over A1 when the latter is off guard. A2, a self‐reported adolescent female personality, usually appears when A comes off duty and feels relaxed at home by herself or with her close friends who know of A's dissociative problems. A1 reports that during her work hours, she is on constant guard so as not to be taken over by A2, as A1 is aware that A2 is somewhere in her mind, impatient to wait until A1 comes off duty.

When A began psychotherapy with Dr. D (a female psychiatrist under my supervision), A1 was the primary person to talk to her therapist. However, as the therapeutic relationship developed, A1 became more relaxed and emotionally dependent on Dr. D, and A1 was sometimes taken over by A2 in the middle of the session. This switch started to occur earlier and earlier in the session until A1 could hardly talk to Dr. D. Around this time, MER (18 mg) was initiated for her ADHD symptoms to help improve her work focus. On the day of the first dose of MER, A1 reported that A2's presence was “suddenly gone” from her mind. After A started using MER, she could remain as A1 during her sessions with Dr. D. However, A1 was concerned that her internal world had drastically changed, and decided to temporarily suspend the use of MER. As a result, A2 soon returned, and A began to take MER “as needed” instead of taking it regularly.

B is a middle‐aged male with DID who experienced a severe trauma in his childhood. Apparently, his father also had DID and repeatedly abused him sexually and physically in the dissociative context, of which his father remained largely unaware of. This may have been due to B's inattention and hyperactivity (B was diagnosed with ADHD in childhood) which irritated his father. B developed several personality states very early in childhood. Nonetheless, B achieved reasonable social adaptation and worked in a local grocery store while living with his male partner. B states that sometimes two or three personalities are mixed or rapidly switching inside him, resulting in frequent memory lapses which considerably impair his job performance. Occasionally, B enters a trans‐like state during his medical visits to me, where he is no longer aware of who and where he is. In that trans‐like state, B states that he might easily switch to a very aggressive personality if he is verbally abused by his male partner. After initiating MER for B's untreated ADHD in order to improve his job performance (initially 18 mg per day, then gradually increased to 56 mg), B reported that it helped him stay alert and much less confused about who he is and the tasks he is engaged in. His violent episodes quickly decreased as chances to switch to the aggressive personality were reduced.

C is a young male university student in his early twenties with occasional dissociative episodes. He was also diagnosed with ADHD during childhood, with inattentiveness and hyperactivity. At the age of 17, when he was taking a written examination for a university, he suddenly felt “foggy” and unable to think or write. He then “came to” just before the end of his examination, and he failed it as was expected. C was examined neurologically at a local hospital, and no organic etiology was found. Finally, C was diagnosed with depersonalization‐derealization disorder (DDD, DSM‐5) [5], in addition to ADHD, by a local psychiatrist who referred C to me. C continued to demonstrate similar trans‐like episodes lasting for several minutes, particularly when he was stressed. After graduation, C managed to get a temporary job as floor staff in a restaurant in the evening but continued to have trans‐like dissociative episodes as well as inattentiveness and forgetfulness, which were considered to be due to his ADHD. When MER (18 mg/day) was administered to help him cope with his work, the frequency of his trans‐like episodes markedly diminished and his job performance improved significantly.

## 3 Discussion

This report is based on observations of patients with DID and other dissociative disorders as well as comorbid ADHD. Patients' DID diagnosis is based on the DSM‐5 criteria and adult ADHD diagnosis is made independently to that of DID, based on the DSM‐5 criteria and the ASRS‐v1.1 checklist, and MER was administered at various stages of their treatment course. Comorbidity of DID and ADHD is not generally discussed in DSM‐5 or ICD‐11, nor in the literature with some exception [5].

The administration of MER strictly followed the Japanese Concerta Circulation Management Committee guidelines.

Many of these patients showed a fair to good response, with improved ADHD symptoms as well as some side effects such as irritation, nausea, and headache. However, unexpectedly, some patients responded remarkably with improved/altered dissociative symptoms. Administration of MER (18 ~ 54 mg) appears to be especially effective for those who tend to be in a state in which a couple or more personalities are in conflict and vying for conscious control. MER appears to increase the threshold separating different personalities and reduce the level of trans‐like or depersonalization symptoms.

These findings may be generalizable besides the three cases described in the case presentation. Approximately 15% ~ 20% of my DID cases have a concurrent diagnosis of ADHD and I have observed favorable to good responses with MER in a significant minority of them.

This favorable effect on dissociative symptoms might not be limited to MER; psychostimulants in general and atomoxetine may share similar results, based on my anecdotal experience. One of my patients, a male corporate employee with DID who is on modafinil for his sleep disturbances, reported a similar effect on his “capacity to stay as himself” while he is on duty. Another patient with DDD demonstrated improved symptoms with atomoxetine (50 mg/day) administered for adult ADHD symptoms.

The opposite may apply to medications classified as “downers” such as benzodiazepine and other GABAergic medications, as well as alcohol. These can induce a dissociative or trance‐like state and may promote personality shifts in patients with DID who appear to suffer a reduction in the threshold separating personalities. In psychiatric literature, certain authors caution against the use of benzodiazepines for dissociative disorders as they may worsen symptoms [1].

Even individuals without dissociative disorders can exhibit a trans‐like state with disinhibited eating behaviors after intake of sleep inducers such as triazolam and zolpidem. Needless to say, the so‐called “black out” dissociative state is so common among those who consume excessive alcohol and become amnestic about their aberrant and uncharacteristic behaviors, and this can be understood in this context of this paper.

The neurobiological mechanisms of dissociative symptoms are far from being clarified and more research is needed to determine the best psychopharmacological treatment. The relationship between dissociative symptoms and psychostimulant medications discussed in this report can aid future clinical research in this area.

## Author Contributions

Kenichiro Okano was the primary care physician of the case B, C, and clinical supervisor of Dr. D, the primary physician of the case A. Dr. D gave her approval for the final version to be submitted.

## Ethics Statement

This study was conducted according to the principles of the Declaration of Helsinki.

## Consent

Informed written consent was obtained from the patient for publication of the report.

## Conflicts of Interest

The author declares no conflicts of interest.

## Data Availability

Data supporting this study are included within the article.
